# A novel error correction protocol for continuous variable quantum key distribution

**DOI:** 10.1038/s41598-021-90055-3

**Published:** 2021-05-17

**Authors:** Kadir Gümüş, Tobias A. Eriksson, Masahiro Takeoka, Mikio Fujiwara, Masahide Sasaki, Laurent Schmalen, Alex Alvarado

**Affiliations:** 1 Department of Electrical Engineering, Eindhoven University of Technology, Eindhoven, 5600MB The Netherlands; 2 National Institute of Information and Communications Technology (NICT), 4-2-1 Nukui-kitamachi, Koganei, Tokyo 184-8795 Japan; 3 Karlsruhe Institute of Technology, Communications Engineering Lab, 76131 Karlsruhe, Germany; 4 Infinera, Fredsborgsgatan 24, 117 43 Stockholm, Sweden

**Keywords:** Electrical and electronic engineering, Quantum physics

## Abstract

Reconciliation is a key element of continuous-variable quantum key distribution (CV-QKD) protocols, affecting both the complexity and performance of the entire system. During the reconciliation protocol, error correction is typically performed using low-density parity-check (LDPC) codes with a single decoding attempt. In this paper, we propose a modification to a conventional reconciliation protocol used in four-state protocol CV-QKD systems called the multiple decoding attempts (MDA) protocol. MDA uses multiple decoding attempts with LDPC codes, each attempt having fewer decoding iteration than the conventional protocol. Between each decoding attempt we propose to reveal information bits, which effectively lowers the code rate. MDA is shown to outperform the conventional protocol in regards to the secret key rate (SKR). A 10% decrease in frame error rate and an 8.5% increase in SKR are reported in this paper. A simple early termination for the LDPC decoder is also proposed and implemented. With early termination, MDA has decoding complexity similar to the conventional protocol while having an improved SKR.

## Introduction

Data security plays a vital role in communications for ensuring that a potential adversary or eavesdropper (Eve) is incapable of gaining access to sensitive information. It is theoretically possible to secure the sensitive information by using encryption^1^. In encryption, a secret key is used to encrypt the information, this encrypted information can only be recovered if it is decrypted using the same secret key. In order for the transmitter (Alice) and the receiver (Bob) to securely exchange information they both need access to the same key, without Eve having any knowledge regarding the secret key. To get the same key, Alice and Bob will exchange keys with each other. It is of the utmost importance that the key exchanging process is completely secure to prevent Eve from gaining knowledge on the key.

Quantum Key Distribution (QKD) is a method of sharing secret keys between Alice and Bob in a secure manner^2^. There are two main variants of QKD, namely discrete-variable (DV)^3^ and continuous-variable (CV)^4^ QKD. In DV-QKD systems, the measurements of the quantum states are done using specialized hardware, e.g., single photon counters. CV-QKD systems, however, use existing technology currently used in telecommunication systems for the measurement. Because CV-QKD does not require such specialized hardware, it is easier to implement into the current telecommunication network when compared to DV-QKD. However, the downside is that error correction in CV-QKD is more complex when compared to DV-QKD^5^. In addition to this, compared to DV-QKD, there has been less research on reconciliation for CV-QKD. For these reasons, we will focus on CV-QKD in this paper. A detailed description of the CV-QKD systems is given in the “Methods” section.

Because of the potential advantages CV-QKD, it has been gaining a lot of attention recently. Joint propagation of CV-QKD has been implemented both with on-off keying signals^6^ and unmodulated classical intensity carriers^7^. Co-propagation of CV-QKD with 56 100 Gb/s coherent classical channels has been shown^8^. Furthermore, co-propagation with 100 wavelength division multiplexing channels with a total data rate of 18.3 Tb/s has been demonstrated over a period of 24 h^9^. For implementations of full CV-QKD systems, secret key rates (SKR) of 5.77 kbps have been reported over distances of 50 km^10^. Additionally, CV-QKD over a distance of 200 km was demonstrated by controlling excess noise and using highly efficient reconciliation procedures with an SKR of 6.2 bps^11^. Recently, CV-QKD has also been implemented on on cost effective silicon photonics chips^12^.

One of the biggest challenges in CV-QKD is the error correction as part of the reconciliation. Because of the very low signal-to-noise ratio (SNR) operating range of the system, error correcting codes with large block sizes and low rates, which require computationally complex decoding algorithms , are necessary for the error correction^13^. Due to these requirements, reconciliation is a major bottleneck of the entire CV-QKD system in regards to both speed and hardware complexity^14^. The performance of reconciliation also heavily influences the performance of the CV-QKD system. For backgrounds and details on the reconciliation process, we refer the interested reader to the “Methods” section (Figs. 6, 7) and references therein.

The types of codes that are most often used during the reconciliation are low-density parity-check (LDPC) codes^4,15^ or more specifically, multi-edge type (MET) LDPC codes^16^. Recently, other error correction codes, such as quasi-cyclic LDPC codes^14^, polar codes^17^, raptor codes^18^ and spatially coupled codes^19^ have been studied, however, we will use MET-LDPC codes in this paper as it is the most commonly used type of code for CV-QKD systems. In the “Methods” section we give more detail on the MET-LDPC codes used.

The SKR is the most important measure of the performance of the reconciliation, representing the rate at which secret keys can be exchanged. The SKR is influenced by the error correction, namely by the rate of the code ($R_c$) and its frame error rate (FER). In order to maximize the SKR during reconciliation, it is preferred that the rates of the codes operate as closely as possible to the capacity, and that the FER is as low as possible. Current reconciliation protocols used for CV-QKD operate with fairly high FERs, but with relatively high $R_c$. Optimizing the trade-off the FER and $R_c$, and therefore increasing the SKR, is the main motivation for this paper.

In this paper, we propose a new reconciliation protocol, called the multiple decoding attempts (MDA) protocol, which is a modification of the one-dimensional reconciliation protocol as described in Ref.^14^ which we will refer to as the conventional protocol throughout the rest of this paper. MDA, as the name implies, involves multiple decoding attempts, with bits being revealed in between attempts. In order to ascertain the performance in regards to SKR of MDA we implement a basic version of it and compare it to the conventional protocol. Numerical results show that MDA offers gains of up to 8.5% in terms of SKR for a typical CV-QKD link.

## Results

### Conventional reconciliation protocol

The SKR is a measure for the performance of the QKD system. For the conventional protocol the SKR is given by^20^

1 $$\begin{aligned} \text {SKR} = (1-\text {FER})(\beta I_{AB}-\chi _{BE}). \end{aligned}$$

Here, $I_{AB}$ is the mutual information between Alice’s and Bob’s measured outcomes, $\chi _{BE}$ is the Holevo information, a measure for the amount of information Eve has on the secret keys, and $\beta$ is the reconciliation efficiency. The reconciliation efficiency is defined as $\beta = \frac{R_c}{I_{AB}}$ and represents the gap between $R_c$ and $I_{AB}$. This gap exists because, in practice, the codes used in QKD systems operate with $R_c < I_{AB}$. Typically, there is a trade-off between $\beta$ and FER, which affects the SKR in Ref.1: the higher $\beta$ becomes, the higher the FER. Ideally, the error correcting code should operate at a high $\beta$ with a low FER.

**Figure 1:**
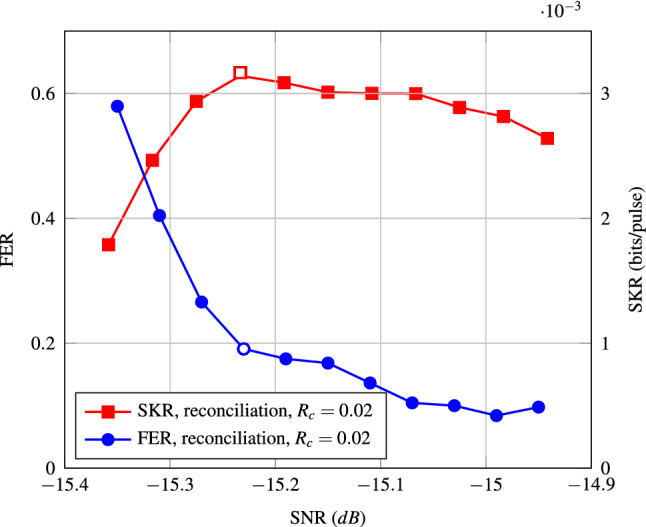
FER and SKR of the conventional protocol over a range of different SNRs for an LDPC code with $R_c = 0.02$. The points corresponding to the optimal SKR are shown with white markers.

Figure 1 shows the FER and SKR for the conventional protocol against the signal to noise ratio (SNR) for a code with $R_c = 0.02$ and 500 decoding iterations. In this paper, we consider CV-QKD with quadrature-phase-shift-keying (QPSK) and heterodyne detection. See “Methods” for the details of the CV-QKD protocol and its SKR analysis. Details on the parameters used in the simulation are given in the simulation set-up section of “Methods”. As the figure shows, for this fixed code rate, the optimal SNR in terms of SKR is around $-15.23$ dB, which corresponds to a $\beta$ of 0.939, as $I_{AB} = 0.0213$ at this SNR. All simulations performed in the rest of this paper are done at $\text {SNR} = -15.23$ dB (white markers). In the next section, we will propose a new protocol which improves the SKR presented in Fig. 1.

### Multiple decoding attempts reconciliation protocol

**Figure 2:**
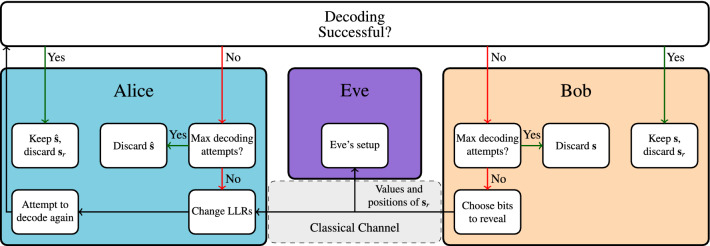
An overview of MDA. After every unsuccessful decoding attempt, Bob reveals bits to Alice, after which Alice will attempt to decode again. This goes on until either the decoding is successful or the maximum amount of decoding attempts has been reached.

In order to improve the SKR without a significant increase in decoding complexity, we propose the new MDA protocol. An overview of MDA is given in Fig. 2. In MDA, we first attempt to decode the codeword in the same manner as in the conventional protocol. After this decoding attempt, we check whether the codeword has been decoded successfully. If so, we keep the codeword and move on to privacy amplification. If not, Bob will choose some information bits to reveal to Alice. This effectively lowers the code rate, and therefore increases the probability of successfully decoding the codeword. The information bits to be revealed are chosen randomly, as revealing bits in this manner does not cause any issues in regards to security, as this is equivalent to the *sp*-protocol already used in reconciliation^21,22^, except that the bits are revealed after a decoding attempt. In the Discussion we mention some other bit revealing strategies which we had considered. Alice adjusts her LLRs based on these revealed bits and attempts to decode again. An important thing to note here is that Alice continues decoding from where she left off after the previous decoding attempt. More information on how exactly this is done is given in the LDPC coding and decoding section in the “Methods”. We then check once again whether the decoding was successful, and we continue with MDA until either the codeword was decoded successfully, or the maximum amount of decoding attempts has been reached. Any bits that were revealed during MDA are discarded when passing on the information bits to privacy amplification, as Eve knows the values of these bits.

MDA is somewhat reminiscent of Raptor^18^ codes, in that extra information on the codeword is revealed after a failed decoding attempt.

The SKR of MDA is a modified version of Eq. (1). In particular, let $\text {FER}_i$ and $\beta _i$ be the frame error rate and reconciliation efficiency for the *i*th decoding attempt and *n* the number decoding attempts. Then it can be shown that

2 $$\begin{aligned} \text {SKR} = (1-\text { FER}_1)(\beta _1 I_{AB}-\chi _{BE}) + \sum _{i = 2}^n (1-\text { FER}_i)(\beta _i I_{AB}-\chi _{BE})\prod _{j = 1}^{ i-1}\text {FER}_j. \end{aligned}$$

Essentially, the SKR of MDA in Eq. (2) is an addition of the SKRs of all decoding attempts. In Eq. (2), the SKR of each decoding attempt is multiplied by the FERs of all previous attempts. This multiplication of FERs represents the fraction of codewords that have not yet been correctly decoded given that the previous decoding attempts were unsuccessful. So for each additional decoding attempt, the maximum SKR that can be gained decreases. Therefore, the SKR resulting from the transmission of a single codeword will never be lower than that of the conventional protocol, as the first decoding attempt would use a code with the same reconciliation efficiency and FER as that of the conventional protocol. Furthermore, the average amount of decoding iterations needed for MDA is

3 $$\begin{aligned} {\overline{D}} = D_{1} + \sum _{i=2}^{n}D_i\prod _{j = 1}^{i-1}\text {FER}_j, \end{aligned}$$

where $D_{i}$ is the maximum amount of iterations for the *i*th decoding attempt. The total amount of decoding iterations that were used for the decoding of a particular codeword depends on the amount of decoding attempts necessary to decode it. As the amount of decoding iterations used differs for each codewords, we have decided to introduce ${\overline{D}}$, which is simply an average over the amount of decoding iterations needed per codeword. We will use Eqs. (2) and (3) to calculate the SKR and the average decoding iterations for MDA in the remainder.

**Figure 3:**
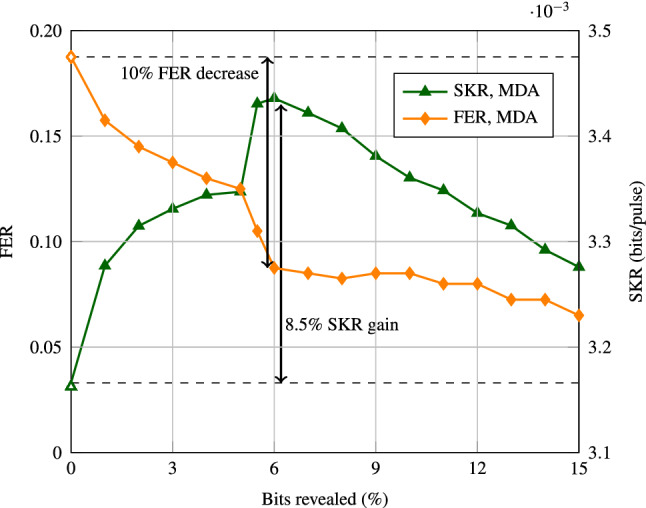
FER and SKR of MDA with $n=2$. The rate $R_c$ of the first code is 0.02. The rate of the second code used depends on the amount of bits revealed, where zero bits revealed (white markers) is equivalent to the conventional protocol. The simulations were performed at $\text {SNR} = -15.23$ dB.

As a proof of concept, we implemented the simplest version of this new protocol ($n=2$). We first performed a sweep over the amount of bits to be revealed for the second decoding attempt, assuming 400 decoding iterations per decoding attempt, i.e., $D_i = 400$. As before, all relevant parameters are mentioned in the simulation set-up section of “Methods”. Figure 3 shows the FER and SKR against the percentage of bits revealed, with 0% bits revealed being the conventional protocol (see Fig. 1). Figure 3 shows that revealing 6% of the information bits, which corresponds to a code with rate $R_c = 0.0188$ and $\beta = 0.883$, gives the largest increase in SKR. The increase is 8.5%, and comes with a 10% decrease in FER. It is interesting to note that there is a jump between 5% and 6% bits revealed. This jump will be discussed in the “Discussion” section.

Figure 3 shows that MDA outperforms the conventional one in regards to SKR. However, for this figure, the amount of decoding iterations is quite large, with ${\overline{D}} = 480$. In order to see how well MDA compares to the conventional one for similar decoding complexity, we have simulated both the conventional and MDA for a range of average decoding iterations. Recall that the amount of decoding iterations per decoding step is the same for both decoding attempts.

**Figure 4:**
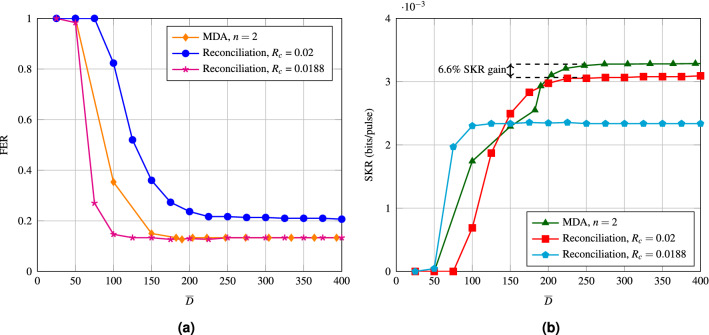
(**a**) FER of MDA compared to an LDPC code with $R_c$ = 0.02, and to an LDPC code with $R_c$ = 0.0188 as a function of the average amount of decoding iterations. All the simulations were performed at an SNR of $-15.23$ dB. (**b**) SKR of MDA compared to an LDPC code with $R_c$ = 0.02, and to an LDPC code with $R_c$ = 0.0188 as a function of the average amount of decoding iterations. All the simulations were performed at an SNR of $-15.23$ dB.

Figure 4 compares both the FER and the SKR of MDA to the conventional one. The FER of MDA converges to the error floor of the $R_c$ = 0.0188 code (6% of bits revealed) for high enough ${\overline{D}}$, which suggests that the FER of MDA is lower bounded by the error floor of the lowest rate code used in the protocol. The right side of the figure shows the SKR, where MDA is shown to outperform the conventional one after roughly 190 decoding iterations. This crossing point is before the SKR curve of the conventional protocol levels off, which is at around 225 iterations. MDA levels off at around 250 iterations. The conventional protocol with the rate $R_c = 0.0188$ code performs the best out of the three until 140 iterations, although in general for the conventional protocol more than 140 decoding iterations are used^14,15^.

### Early termination

Early termination (ET) is a method often used during error correction to reduce the processing latency (i.e., ${\overline{D}}$). Due to the use of multiple decoding attempts, ET is expected to have a greater impact on the performance of MDA when compared to the conventional protocol. This is because with the additional decoding attempts, we continue the decoding from where we left off after the previous decoding attempt. So the decoder should be closer to converging at the start of the current decoding attempt, and should therefore require fewer decoding iterations when compared to the previous decoding attempt. We have implemented an ET protocol that is inspired by the sign-change-ratio method used for Turbo codes^23^. During decoding, we track the changes in the output bits of the recovered codeword over the iterations. If, for a certain amount iterations (in our case 5), we do not sense any change in the output bits of our decoder, we assume that the decoder has converged. This approach proves to be more effective than the ET based on the parity check equations when working with applications with a high FER. ET based on the parity check equations can only detect correctly decoded codewords. Our method, however, can also perform ET on codewords where decoding is not successful, which is especially useful for MDA and CV-QKD in general.

**Figure 5:**
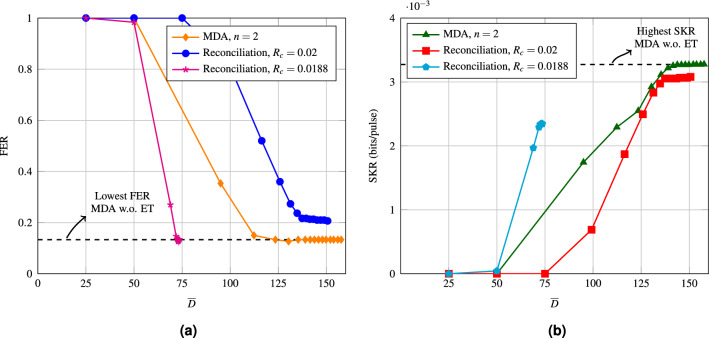
(**a**) FER of MDA compared to an LDPC code with $R_c$ = 0.02, and to an LDPC code with $R_c$ = 0.0188 as a function of the average amount of decoding iterations with ET. All the simulations were performed at an SNR of $-15.23$ dB. (**b**) SKR of MDA compared to an LDPC code with $R_c$ = 0.02, and to an LDPC code with $R_c$ = 0.0188 as a function of the average amount of decoding iterations with ET. All the simulations were performed at an SNR of $-15.23$ dB.

Figure 5 shows the results obtained by ET for both the conventional protocol and MDA. Figure 5 shows that with ET the FER and SKR of the proposed are the same as without ET, which suggests that ET has little to no effect on the performance. With ET, the SKR levels off at 131 iterations for the conventional protocol and at 139 iterations for the proposed one. Which means that ET has a slightly larger effect on when then SKR levels off for MDA (44.4% decrease in ${\overline{D}}$) when compared to the conventional protocol (41.7% decrease in ${\overline{D}}$), confirming that the second decoding attempt requires fewer decoding iterations. With ET, MDA outperforms the conventional protocol with the $R_c = 0.02$ code for any amount of decoding iterations. The conventional protocol with the $R_c = 0.0188$ code performs the best out of the three until about 125 iterations.

### Latency of MDA

One additional factor that has to be taken into account when comparing the conventional protocol with MDA is the additional delay introduced by Bob divulging information bits to Alice. Every additional decoding attempt requires a back-and-forth two-way communication between Alice and Bob, and therefore introduces a transmission delay. This transmission delay is approximately equal to the transmission delay caused by starting the reconciliation over again with a new codeword, as both require a back-and-forth between Alice and Bob. So the transmission delay caused by MDA when we attempt to decode a codeword twice is equal to when we try to decode two different codewords once each, as this requires the same amount of back-and-forths. So if we want to compare the transmission delay of MDA to that of the conventional protocol, we need to look at how many decoding attempts are required for creating a secret key for both protocols.

We compare the transmission delays of both protocols by means of a numerical example. For the sake of simplicity, we only look at the transmission delays, so any other delays caused by post-processing or the transmission and measurement of quantum states is not taken into account. In privacy amplification, secret keys are generated by hashing a block of length $N_{\text {privacy}}$ bits. This block of length $N_{\text {privacy}}$ is construed from the information bits of the correctly decoded codewords. In order to limit any finite size effects, $N_{\text {privacy}}$ is often chosen in the order of $10^{12}$^14^. For the conventional protocol with $R = 0.02$ and $N = 10^6$, 20,000 information bits are recovered from every correctly decoded codeword. So $10^{12}/20,000 = 5\cdot 10^7$ codewords need to be decoded correctly in order to create one block for the privacy amplification. Looking at our results from Fig. 3, where the conventional protocol has an FER of 19%, the expected number of codewords that need to be transmitted in order to move on to privacy amplification equals $5\cdot 10^7/0.81 = 6.17\cdot 10^7$. Because there is only one decoding attempt in the conventional protocol, the total amount of expected decoding attempts is therefore also equal to $6.17\cdot 10^7$.

For MDA, our results in Fig. 3, where we have $n=2$ decoding attempts, indicate that the FER for MDA equals 19% after the first decoding attempt and 9% after the second decoding attempt. This means that 19% of all codewords move on to the second decoding attempt and that of the correctly decoded codewords, 89% were decoded correctly after the first attempt and 11% after the second attempt. The number of information bits that are retrieved from a codeword is 20,000 for codewords that were correctly decoded after the first attempt and 18,800 for codewords that were correctly decoded after the second attempt. This is because we discard the 6% of information bits that we reveal during MDA, which is equivalent to 1200 bits. On average, that would mean that $0.89\cdot 20,000 + 0.11 \cdot 18,800 = 19,868$ bits are retrieved per correctly decoded codeword. The expected number of codewords that need to be decoded correctly for a single block in the privacy amplification is then equal to $10^{12}/19,868 = 5.03 \cdot 10^7$. With an FER of 9%, the expected number of codewords that need to be transmitted in order to move on to privacy amplification equals $5.03 \cdot 10^7/0.91 = 5.53\cdot 10^7$, 10% less than for the conventional protocol. Of these codewords, 81% ($4.48\cdot 10^7$) require one decoding attempt, and 19% ($1.05\cdot 10^7$) move on to the second decoding attempt, so the total amount of decoding attempts is then equal to $4.48\cdot 10^7 + 2\cdot 1.05\cdot 10^7 = 6.58\cdot 10^7$. This is an increase of only 6.7% in decoding attempts when compared to the conventional protocol. If we assume that each decoding attempt has an equal contribution to the latency, this will be a small increase of 6.7% in latency.

In reality, the difference in total delay between the two protocols will smaller and almost negligible. This is because there are additional delays which we have not taken into account in the analysis: The processing delay caused by the decoder is usually smaller for the second decoding attempt when compared to the first decoding attempt, as the second decoding attempt requires fewer decoding iterations. Additionally, for the second decoding attempt, no new quantum states measurements are required unlike in the conventional case when we try to decode a new codeword. As the total amount of codewords that need to be transmitted is 10% less for MDA, this also means that 10% fewer quantum states need to be transmitted and measured. This transmission and measurements of quantum states induces a further delay for the conventional protocol.

## Discussion

A new reconciliation protocol was proposed in this paper. This protocol shows an improvement in SKR over the conventional protocol of up to 8.5% (see Fig. 3). We have only shown one simple implementation of the new protocol, and despite the fact that the parameters still need to be optimized, MDA still outperforms the conventional one. With further research on how the different hyper parameters affect MDA, the gap between them should grow ever larger.

In general MDA works better than the conventional protocol for higher ${\overline{D}}$. For the low ${\overline{D}}$ region the conventional protocol with the lower rate performs the best, and with ET, MDA only overtakes the conventional protocol after 125 decoding iterations. MDA is therefore more suited for CV-QKD systems where decoding latency with ${\overline{D}} > 125$ is allowed.

A very important factor to the performance of MDA is the type of code used. Here, we used low rate MET-LDPC codes. However, the code we used in the paper has a high error floor^14^. Normally, this is not of importance for the reconciliation, as we work around the waterfall region of the codes. For MDA, however, the codes used in subsequent decoding attempts operate in their error floor region. If we were to design codes with lower error floors and use them for MDA, it would lead to a larger increase in SKR.

In another version of MDA we discarded all of the messages within the LDPC decoder for each decoding attempt, effectively restarting the entire decoding process. This, however, proved to be inferior to keeping the messages within the decoder with a new decoding attempt, which is similar to continuing the previous decoding attempt. Other ways to use the messages within the decoder to our advantage need to be further investigated.

Finally, we have also considered different strategies for which bits to reveal. Currently, we randomly choose which bits to reveal. This method leads to a sudden jump in FER, similar to a waterfall region (see Fig. 3). This behavior is under further investigation. We have also tried choosing which bits to reveal based on the LLRs of the information bits at the end of a decoding attempt. This bit revealing strategy gave a significant improvement in regards to performance, however it is very likely that doing this gives away information to Eve and potentially renders the reconciliation less secure. Another method considered was choosing which bits to reveal based on the absolute values of Bob’s measurement outcomes. This is similar to post selection^24^, however, once again, it has not been proven yet that this is has no effect on the security of the reconciliation.

## Methods

### CV-QKD

**Figure 6:**
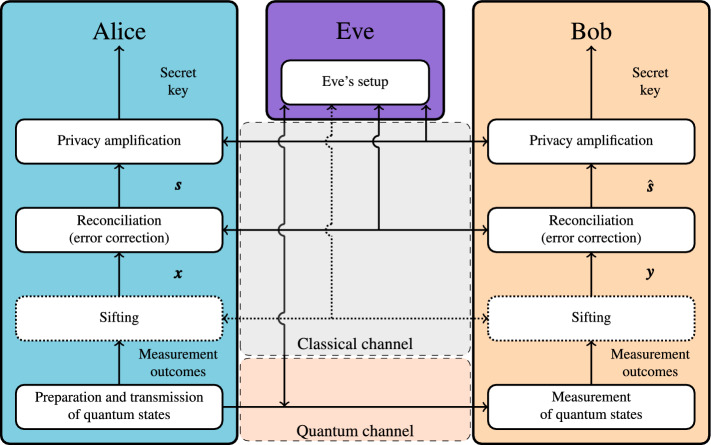
An overview of the CV-QKD protocol based on four different stages; transmission and measurements of quantum states, sifting, reconciliation, and privacy amplification. Eve has full access to classical channel and full control over the quantum channel. The purpose of the protocol is to ensure that at the end Alice and Bob are left with the same secret keys.

The CV-QKD protocol consists of four steps^20^, as shown in Fig. 6: transmission and measurement of quantum states, sifting, reconciliation and privacy amplification. During quantum transmission, Alice prepares quantum states to be transmitted to Bob. The information is modulated onto the amplitude and phase operators of the coherent state and then transmitted to Bob over the quantum channel. Although Gaussian modulation is often used^20^, in this paper we assume that Alice and Bob make use of the four-state protocol^5^, as in systems implemented in real hardware transmitting over large distances, using Gaussian modulation is difficult^25^. Although there is still no unconditional security proof of the four-state protocol, a more recent security analysis has paved the way to a full security proof and has shown that the four state protocol can achieve secret key rates comparable to that of the Gaussian modulation^26^. This, however, does not mean that MDA is restricted to only the four-state protocol. MDA can be used in conjunction with an arbitrary modulation format.

After Bob has measured Alice’s transmitted quantum states, they both move onto sifting. During sifting, depending on whether homodyne (sifting) or heterodyne (no sifting) detection is used, Bob and Alice discard some of their measurement outcomes. Alice and Bob are now left with their sequences of modulation values and measurement outcomes, ${\varvec{x}}$ and ${\varvec{y}}$, respectively. In reconciliation, error correction is performed to ensure that Bob and Alice are left with the same sequence of bits, i.e., ${\varvec{s}} = \hat{{\varvec{s}}}$ (see Fig. 6). During reconciliation, ${\varvec{x}}$ and ${\varvec{y}}$ will be used to demodulate and modulate the bit sequences respectively, which will be used to form the secret key. We will go into more detail about how the reconciliation works later in the “Methods” section. Finally, during the privacy amplification, a hashing function is applied to Bob’s and Alice’s bit sequences. The sequence Alice and Bob are left with at the end of the privacy amplification is the key that can be used for the encryption. Concrete analysis of the privacy amplification is outside the scope of this paper.

### Reconciliation

**Figure 7:**
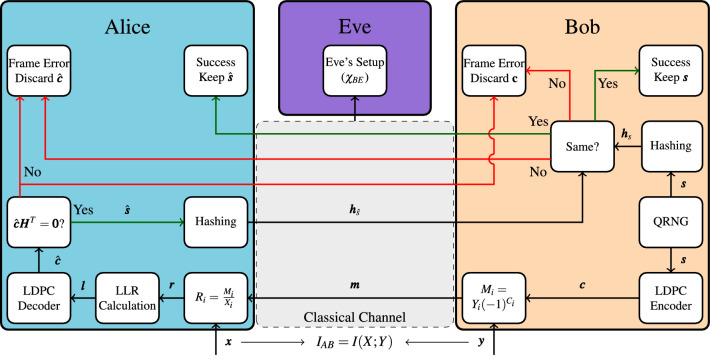
An overview of the one-dimensional reconciliation protocol for CV-QKD considered in this paper. The goal of reconciliation is for Alice and Bob to have the same sequence after error correction (e.g., ${\varvec{s}} = \hat{{\varvec{s}}}$) using their sequences of measurement outcomes (${\varvec{x}}$ and ${\varvec{y}}$) respectively. Eve has full access to the classical channel.

There are two types for reconciliation: forward reconciliation and reverse reconciliation. In forward reconciliation, Alice creates the codeword and Bob attempts to decode it, while in reverse reconciliation the roles are reversed. Reverse reconciliation is the preferred method, as forward reconciliation heavily limits the transmission distance^20^, therefore this paper considers reverse reconciliation. An overview of the reconciliation protocol is shown in Fig. 7.

Because we are assuming the use of a four-state protocol, the reconciliation is one-dimensional. At the start of the protocol, Alice has a sequence of randomly generated modulation values ${\varvec{x}} = (x_1^\text {I}, x_1^\text {Q}, x_2^\text {I}, x_2^\text {Q}, \cdots , x_{N/2}^\text {I}, x_{N/2}^\text {Q})$ to prepare a sequence of *N*/2 QPSK coherent states, where *N* is the block length of the code that will be used for the reconciliation. Here, $x_i^\text {I}$ is the real part and $x_i^\text {Q}$ is the imaginary part of the QPSK modulation for the *i*th transmitted coherent state, respectively. As we make use of the four-state protocol, $x_i$ is a randomly chosen QPSK symbol. In the following, we use the normalized ${\varvec{x}}$ as ${\mathbb {E}}[|X|^2] = 1$.

On the receiver side, Bob has his sequence of measured outcomes of the detected quantum signals as ${\varvec{y}} = (y_1^\text {I}, y_1^\text {Q}, y_2^\text {I}, y_2^\text {Q}, \cdots , y_{N/2}^\text {I}, y_{N/2}^\text {Q})$, where once again I and Q represent the real and imaginary parts of the measured outcomes, respectively, and ${\varvec{y}}$ is also normalized with condition ${\mathbb {E}}[|X|^2] = 1$. During the reconciliation protocol we consider the quantum channel to be an additive white Gaussian noise (AWGN) channel, where ${\varvec{y}} = {\varvec{x}} + {\varvec{z}}$ with ${\varvec{z}} = (z_1^\text {I}, z_1^\text {Q}, z_2^\text {I}, z_2^\text {Q}, \cdots , z_{N/2}^\text {I}, z_{N/2}^\text {Q})$, and $Z_i^\text {I} \sim {\mathscr {N}}(0,\sigma _z^2/2)$ and $Z_i^\text {Q} \sim {\mathscr {N}}(0,\sigma _z^2/2)$ are independent Gaussian random variables. Here, ${\mathscr {N}}$($\mu$,$\sigma ^2$) represents a Gaussian distribution with mean $\mu$ and variance $\sigma ^2$, and $\sigma _z^2$ is the noise variance of $Z_i^I + jZ_i^Q$. Note that upper case letters represent random variables and lower case letters their respective realizations. For ease of use, from now on we will represent ${\varvec{x}}$ as $(x_1, x_2, \cdots , x_N)$, where $x_1 = x_1^\text {I}$, $x_2 = x_1^\text {Q}$, $\cdots$, $x_{N-1} = x_{N/2}^\text {I}$, $x_N = x_{N/2}^\text {Q}$. The same notation applies to ${\varvec{y}}$ and ${\varvec{z}}$.

Bob generates a random sequence of bits ${\varvec{s}} = (s_1, s_2, \cdots , s_{R_c\cdot N})$ of length $R_c\cdot N$ using, for instance, a quantum random number generator (QRNG). Bob then encodes ${\varvec{s}}$ using an LDPC encoder, which results in a codeword ${\varvec{c}} = (c_1, c_2, \cdots , c_N)$. The LDPC codes used during reconciliation are systematic for the sake of the hashing performed later in the reconciliation. Bob modulates ${\varvec{c}}$ using the invertible function

4 $$\begin{aligned} m_i = y_i (-1)^{c_i}, \quad i = 1,2,\ldots , N. \end{aligned}$$

The output ${\varvec{m}}$ of this function is transmitted to Alice over the classical channel (see Fig. 2). We assume that the classical channel adds no errors and that Eve has full access to it.

Alice receives ${\varvec{m}}$ and demodulates it as

5 $$\begin{aligned} r_i = \frac{m_i}{x_i} =\frac{(-1)^{c_i}y_i}{x_i} = \frac{(-1)^{c_i}(x_i+z_i)}{x_i} = (-1)^{c_i} + (-1)^{c_i}\frac{z_i}{x_i}. \end{aligned}$$

The variable $r_i$ in Eq. (5) is effectively the output of a binary input AWGN (BI-AWGN) channel with a signal to noise ratio (SNR) of $\text {SNR} = \frac{1}{\sigma _z^2}$. This BI-AWGN channel allows us to use the very efficient error correction codes compatible with this type of channel. Using ${\varvec{r}}$, Alice calculates the log-likelihood ratios (LLRs) of her received sequence. The LLRs for one-dimensional reconciliation using the four-state protocol are given by

6 $$\begin{aligned} l_i = \log \frac{P(R_i = r_i|C_i = 0)}{P(R_i = r_i|C_i = 1)} = \frac{2R_i}{\sigma _{z}^2}. \end{aligned}$$

Alice attempts to decode the codeword using the LLRs. At the end of her attempt, she is left with an estimate of the original codeword $\hat{{\varvec{c}}}$. To ascertain whether this codeword is correct Alice first calculates the syndrome of $\hat{{\varvec{c}}}$: $\hat{{\varvec{c}}}{\varvec{H}}^T$. If this is not equal to the zero vector, the decoding attempt has failed, and Alice will notify Bob of this failure. They then both discard their codewords and the reconciliation starts anew. If $\hat{{\varvec{c}}}{\varvec{H}}^T = {\varvec{0}}$, it means $\hat{{\varvec{c}}}$ is a valid codeword. This, however, does not imply that $\hat{{\varvec{c}}}$ is equal to ${\varvec{c}}$.

To check whether $\hat{{\varvec{c}}}$ is actually equal to ${\varvec{c}}$, Alice first discards the parity bits of her codeword, leaving her with $\hat{{\varvec{s}}}$. She then applies a universal hashing function on $\hat{{\varvec{s}}}$ and transmits the result of this hashing function (${\varvec{h}}_{{\hat{s}}}$) to Bob. Bob checks the result of his own hashing function (${\varvec{h}}_s$) to that of Alice. If they are not the same, the decoding was a failure and Bob will notify Alice of this and they both discard their codewords. If the hashing results are the same, the reconciliation was successful, and Bob notifies Alice. They can now continue with the privacy amplification.

### LDPC coding and decoding

An LDPC code is defined by its parity-check ${\varvec{H}}$ of size $\hbox {dim}{\varvec{H}}=M\times N$, where $M = (1-R_c)\cdot N$. The codewords belong to the null space of ${\varvec{H}}$. The entry at row *i* and column *j* of ${\varvec{H}}$ is denoted as $H_{i,j}$. Let ${\mathscr {V}}_j = \{i\in \{1,\ldots , M\}: H_{i,j}=1\}$ denote the set containing the positions of nonzero entries in column *j* of ${\varvec{H}}$ and ${\mathscr {C}}_i= \{j\in \{1,\ldots , N\}: H_{i,j}=1\}$ denote the set containing the positions of nonzero entries in row *i* of ${\varvec{H}}$.

The LDPC code used in this paper is an MET-LDPC code with $R_c = 0.02$ code as proposed in Ref.^15^ with degree distributions

7 $$\begin{aligned} \nu ({\varvec{x}}) = 0.0225\cdot x_1^2x_2^{57}x_3^0 + 0.0175\cdot x_1^3x_2^{57}x_3^0 + 0.96\cdot x_1^0x_2^0x_3^1, \end{aligned}$$

and

8 $$\begin{aligned} \mu ({\varvec{x}}) = 0.010625\cdot x_1^3x_2^0x_3^0 + 0.009375\cdot x_1^7x_2^0x_3^0 + 0.6\cdot x_1^0x_2^2x_3^1 + 0.36\cdot x_1^0x_2^3x_3^1, \end{aligned}$$

where $\nu ({\varvec{x}})$ and $\mu ({\varvec{x}})$ are the degree distributions of the variable nodes and the check nodes, respectively^16^. For our simulations, we randomly sample parity check matrices that satisfy these degree distributions. This can be achieved using the graph representation of the code. To each of the *N* columns of ${\varvec{H}}$, we assign a so-called *variable node* and to each of the *M* rows, we assign a so-called *check nodes*. Each node is equipped with (colored) sockets of three different types (type 1, 2, and 3). The multivariate polynomial $\nu ({\varvec{x}})$ defines the nodes and sockets in the following way: the monomial $\nu _i\cdot x_1^{d_{i,1}}x_2^{d_{i,2}}x_3^{d_{i,3}}$ indicates that a fraction $\nu _1$ of the variable nodes are equipped with $d_{i,1}$ sockets of type 1, $d_{i,2}$ sockets of type 2 and $d_{i,3}$ sockets of type 3. Similarly, the monomial $\mu _i\cdot x_1^{d_{i,1}}x_2^{d_{i,2}}x_3^{d_{i,3}}$ indicates that a fraction $\mu _i$ of the *M* check nodes are equipped with $d_{i,j}$ sockets of type *j*. A bipartite graph is formed by randomly connecting an empty variable node socket of type *i* with an empty check node socket of the same type, such that parallel edges are avoided. The socket counts at variable and check nodes match if $R_c = 1-\frac{M}{N}$. The parity-check matrix ${\varvec{H}}$ of the code contains a “1” at row *j* and column *i* if and only if check node *j* is connected to variable node *i* via an edge. Further details on the construction can be found in Ref.^16^ and references therein.

For the decoding of the MET-LDPC codes we use belief-propagation. Two of the most well known algorithms for this are the sum-product algorithm^27^ and the min-sum algorithm^28^. The min-sum algorithm is less complex than the sum-product algorithm, but has a worse error correction performance. Normally, min-sum is often used due to its reduced complexity, but at the low operational SNR of the QKD system, the drop in performance by using it is too significant^29,30^. Because of this, the sum-product algorithm is preferred in QKD systems and is used for our simulations.

In the sum-product algorithm, we iteratively update messages that allow us to improve the reliability of single bits of the codewords. We first initialize the so-called variable node messages with Alice’s calculated LLRs

9 $$\begin{aligned} L(q_{i,j})^{\langle 0 \rangle } = l_i, \qquad \forall i\in \{1,\ldots , N\}, j\in \{1,\ldots , M\}, \end{aligned}$$

where $L(q_{j,i})^{\langle k \rangle }$ denotes the message corresponding to row *i* and column *j* of ${\varvec{H}}$ at decoding iteration *k*. These messages are used to calculate

10 $$\begin{aligned} L(r_{j,i})^{\langle k \rangle } = 2\tanh ^{-1}\left( \prod _{i^\prime \in {\mathscr {C}}_{j}\backslash \{i\}}\tanh \left( \frac{1}{2}L(q_{i^\prime ,j})^{\langle k-1 \rangle }\right) \right) , \qquad \forall j\in \{1,\ldots , M\}, \forall i\in {\mathscr {C}}_i. \end{aligned}$$

These so-called check messages are then used again to update

11 $$\begin{aligned} L(q_{i,j})^{\langle k \rangle } = l_i + \sum _{j^\prime \in {\mathscr {V}}_{i}\backslash \{j\}} L(r_{j^\prime ,i})^{\langle k \rangle }, \qquad \forall i\in \{1,\ldots , N\}, \forall j\in {\mathscr {V}}_j. \end{aligned}$$

The output LLRs are also immediately calculated using the check node messages

12 $$\begin{aligned} L(Q_i)^{\langle k \rangle } = l_i + \sum _{j\in {\mathscr {V}}_i}L(r_{j,i})^{\langle k \rangle }. \end{aligned}$$

The output bit ${\hat{C}}_i$ is a 0 if $L(Q_i) \ge 0$ and a 1 if not.

In MDA, after a failed decoding attempt, Bob reveals the value of some of the information bits to Alice. Now that Alice knows the values of these bits, she sets the *l* these bits to an arbitrarily large value, $10^{10}$ if the bit is a zero, $-10^{10}$ if it is a one. We call this new sequence of LLRs ${\varvec{l}}^{new}$. When Alice attempts to decode again, she does not start over from the beginning, but continues decoding from where she left off, with the only difference being that $l_i$ in Eqs. (11) and 12 is now replaced with $l_i^{new}$.

### Simulation set-up

We have 300 simulation instances for every point in our figures. Our protocol employs the QPSK modulation with coherent state $|\alpha e^{i(k+1)\pi /4}\rangle$ ($k=0,1,2,3$) and heterodyne detection (i.e. no sifting). We derive the key rate based on the security analysis in Refs.^25,31,32^, where the Holevo information between Bob and Eve for the QPSK signal is bounded by that for the Gaussian modulation signal. Though this approach has limitation on the security in the sense that it needs to assume linear channels, it makes the analysis of the quantum part rather simple. We use Eqs. (7) and (9) from^32^ for the calculation of $I_{AB}$ and $\chi _{BE}$ respectively. We use the following parameters: variance for the Gaussian modulation $V_{A} = 2|\alpha |^2 = 0.5$, detection efficiency $\eta _{eff} = 0.5$, channel excess noise $\epsilon = 0.01$ and detector’s electric noise $v_{el} = 0.1$. The value of channel transmittance *T* varies depending on the SNR of the BI-AWGN channel, which is defined in Reconciliation part, with the relation

13 $$\begin{aligned} \mathrm{SNR} = \frac{1}{\sigma _z^2} = \frac{T \cdot V_A}{T \cdot \epsilon + 2\frac{1 + v_{el}}{\eta _{eff}}}. \end{aligned}$$

For example, $T = 0.131$ for $\text {SNR} = -15.23$ dB. We assume that we work with a quantum channel that has a transmission loss $\alpha$ equal to 0.2 dB/km (standard single mode fiber). As the transmittance is related to the distance in kilometers *d* by $T = 10^{-\frac{\alpha d}{10}}$, an SNR of $-15.23$ dB corresponds to a distance of approximately 84 km. All simulations were implemented in MATLAB®.
